# The Impact of Serum Uric Acid on the Progression of Amyotrophic Lateral Sclerosis in Adults Aged 18 and Older: A Systematic Review

**DOI:** 10.7759/cureus.42312

**Published:** 2023-07-22

**Authors:** Sally Hussein, Shravya Pingili, Vijaya Krishna Makkena, Arturo P Jaramillo, Babatope L Awosusi, Javaria Ayyub, Karan Nareshbhai Dabhi, Namra V Gohil, Nida Tanveer, Pousette Hamid

**Affiliations:** 1 Internal Medicine, California Institute of Behavioral Neurosciences and Psychology, Fairfield, USA; 2 Medicine/Surgery, Kakatiya Medical College, Hyderabad, IND; 3 Medicine/Surgery, Osmania Medical College, Hyderabad, IND; 4 General Practice, California Institute of Behavioral Neurosciences and Psychology, Fairfield, USA; 5 Pathology and Laboratory Medicine, California Institute of Behavioral Neurosciences and Psychology, Fairfield, USA; 6 Internal Medicine, Medical College Baroda, Vadodara, IND; 7 Internal Medicine, University of Louisville, Louisville, USA; 8 Neurology, California Institute of Behavioral Neurosciences and Psychology, Fairfield, USA

**Keywords:** als, biomarker, urate level, uric acid, disease progression, amyotrophic lateral sclerosis

## Abstract

We have conducted this review to see if serum uric acid (UA) is associated with slowing amyotrophic lateral sclerosis (ALS) progression in adult patients who are at least 18 years old. Understanding the effects of this biomarker for future use is critical because of its easy accessibility. This systematic review paper examined five previous years of recent studies and reports, published in English and limited to human investigations from the Cochrane, PubMed, and Google Scholar databases. Using instruments for assessing the eligibility and quality of systematic and narrative reviews, we narrowed our search to 11 reports that show evidence of a positive association between high blood uric acid and the progression of ALS. However, this claim still needs confirmation by future studies to confirm that possibility. The results of this systematic review may provide a strong foundation for future studies on this biomarker, demonstrating the significance of blood uric acid levels in ALS and highlighting the necessity of using that biomarker to track the disease's progression.

## Introduction and background

The deadly neurodegenerative condition known as amyotrophic lateral sclerosis (ALS) affects the central nervous system (CNS) and causes upper and lower motor neuron degeneration resulting in motor symptoms. Hyperreflexia in segmental regions of atrophying muscle, unaccompanied by sensory disruption, is a hallmark of ALS that is often absent in other neurodegenerative diseases. The clinical picture, which must involve motor deterioration and may include cognitive and behavioral symptoms, depends on the location and degree of the degeneration. Presentation, development, and survival are all highly variable. Several different subtypes of ALS include progressive bulbar palsy, limb onset, progressive muscular atrophy, and upper motor neuron (UMN) predominant [1].

According to reports, up to 45% of ALS patients experience cognitive impairment at some point during their illness. There could be an impact on many neuropsychological domains. The most frequent deficits are in verbal fluency, social cognition, and executive function [2]. Comprehensive neuropsychological tests have formed the foundation for diagnosing cognitive impairment [3]. However, the physical limitations experienced by ALS patients can pose challenges when it comes to completing these assessments. Timely detection of cognitive impairments in individuals with ALS is of utmost importance, as these impairments have the potential to impact survival rates [4]. Conversely, blood biomarkers can offer a convenient and accessible alternative when available [5].

Oxidative stress plays a role in the pathogenic events that result in neuronal destruction in ALS [6]. Numerous studies on ALS and other neurodegenerative disorders have focused on the neuroprotective impact of plasma uric acid (UA) as an antioxidant [7]. According to two case-control studies, ALS patients had much lower plasma uric acid than healthy controls, which was linked to a faster rate of disease deterioration [8]. Two randomized clinical trials showed that ALS patients with greater baseline uric acid levels had longer survival advantages [9]. Additionally, uric acid levels were negatively correlated with disease stages, which was considerably reversed as the disease advanced [10]. In addition, a prospective study with 319,617 participants discovered that uric acid level was inversely connected to ALS risk in healthy persons [11]. Complex pathophysiological factors, such as abnormal RNA metabolism, impaired DNA repair, dysfunctional mitochondria, and oxidative stress, all play a role in the onset and progression of ALS [12]. Therefore, serum uric acid is currently receiving more attention as a potential biomarker of ALS risk and development [13].

In this systematic review, we aimed to highlight the role of serum uric acid in the progression of the disease, as well as any positive or negative associations that suggest future applications of this biomarker.

## Review

Methodology

**Figure 1:**
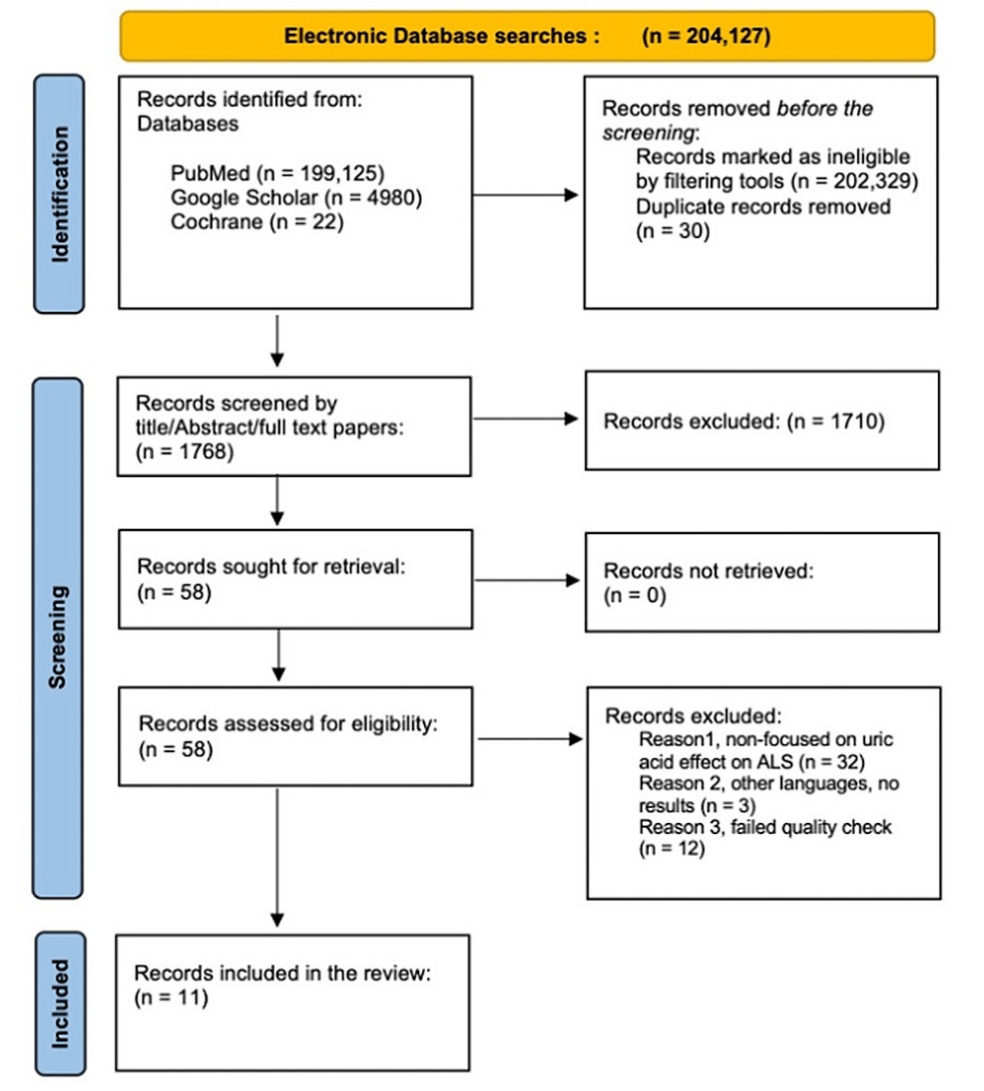
PRISMA 2020 flow diagram for the included studies PRISMA: Preferred Reporting Items for Systematic Reviews and Meta-Analysis

On April 19, 2023, we conducted our systematic review using Preferred Reporting Items for Systematic Reviews and Meta-Analysis (PRISMA) (2020). Figure 1 shows the PRISMA flowchart, which shows the process of the selection and exclusion of the publications used [14].

Data Sources

The online databases used for data collection include PubMed, Google Scholar, and Cochrane Library.

Search Strategy

We used the three databases to collect the required studies for that systematic review. We included clinical trials, narrative reviews, systematic reviews, observational studies, and randomized clinical trials to collect data relevant to the effect of uric acid levels on the progression of the neurological disorder ALS, using common keywords such as amyotrophic lateral sclerosis, ALS, and Lou Gehrig's disease. We used Medical Subject Headings (MeSH) search strategies for related studies on PubMed to capture more articles discussing our research question. Table 1 includes research results for each concept. We used filters such as publication year, human studies, and English articles as primary filters and removed duplicates. We then applied exclusion and inclusion criteria for the remaining papers, excluding those that did not meet our requirements. We evaluated the remaining articles for quality check purposes. After evaluation, we selected only 11 papers that showed high quality and low risk of bias for inclusion in the systematic review, the final number of selected articles in our systematic review that met the selection criteria.

**Table 1:** Initial search results (keywords and MeSH keywords) Results were listed for each keyword/MeSH ALS: amyotrophic lateral sclerosis, MeSH: Medical Subject Headings

Keywords/MeSH	PubMed results	Google Scholar results	Cochrane Library results
Amyotrophic lateral sclerosis OR ALS OR Lou Gehrig's Disease OR (("Amyotrophic Lateral Sclerosis/blood" (Majr) OR "Amyotrophic Lateral Sclerosis/complications" (Majr) OR "Amyotrophic Lateral Sclerosis/diagnosis" (Majr) OR "Amyotrophic Lateral Sclerosis/mortality" (Majr) OR "Amyotrophic Lateral Sclerosis/physiopathology" (Majr))) OR ("Amyotrophic Lateral Sclerosis/blood" (Mesh:NoExp) OR "Amyotrophic Lateral Sclerosis/complications" (Mesh:NoExp) OR "Amyotrophic Lateral Sclerosis/diagnosis" (Mesh:NoExp) OR "Amyotrophic Lateral Sclerosis/mortality" (Mesh:NoExp) OR "Amyotrophic Lateral Sclerosis/physiopathology" (Mesh:NoExp))	165	434,000	22
Uric acid OR serum urates OR (("Uric Acid/adverse effects" (Majr) OR "Uric Acid/blood" (Majr) OR "Uric Acid/cerebrospinal fluid" (Majr) OR "Uric Acid/metabolism" (Majr) OR "Uric Acid/pharmacokinetics" (Majr) OR "Uric Acid/poisoning" (Majr) OR "Uric Acid/toxicity" (Majr))) OR ("Uric Acid/adverse effects" (Mesh:NoExp) OR "Uric Acid/blood" (Mesh:NoExp) OR "Uric Acid/cerebrospinal fluid" (Mesh:NoExp) OR "Uric Acid/metabolism" (Mesh:NoExp) OR "Uric Acid/pharmacokinetics" (Mesh:NoExp) OR "Uric Acid/poisoning" (Mesh:NoExp) OR "Uric Acid/toxicity" (Mesh:NoExp))	20,036	20,800	12
Progression OR advancement OR development OR ("Disease Progression" (Majr)) OR "Disease Progression" (Mesh:NoExp)	189,675	6,020,000	226

Inclusion Criteria

We included studies published within the past five years in English starting from January 2018 to the date we started the research. Only human studies were included. Our systematic review included traditional, systematic, and observational studies and meta-analyses. Inclusion criteria included ALS patients aged 18 and above. We ensured that our approach to data collection was both ethical and legal.

Exclusion Criteria

We eliminated animal studies. Studies not published in the English language and those older than five years were excluded. Studies of patients under 18 years confirmed with other neurological disorders leading to muscle paralysis also were excluded.

Quality Assessment Tools

We evaluated the remaining articles for a quality check using the assessment of multiple systematic reviews (AMSTAR) questionnaires as a quality assessment tool for systematic reviews and meta-analysis records. We used the Newcastle-Ottawa Scale questionnaire for observational studies and the Scale for the Assessment of Narrative Review Articles (SANRA) scale for the traditional review. Low-quality records were excluded.

Data Collection

Data collection was captured from the final articles after quality assessment.

Results

Following the database research, 204,127 reports were identified from PubMed, Google Scholar, and Cochrane. After placing specific filters and removing duplicates (n=30), 1,768 reports were included in the screening procedure. Screening by titles and abstracts excluded 1,710 as non-relevant to our research topic, and 58 articles underwent an eligibility check as a pre-final step. After reviewing 58 reports per inclusion and exclusion criteria, we evaluated the remaining articles for quality assessment as a final step in choosing the final reports. We included 11 articles for data extraction and excluded 12 for low-quality aspects (Figure 1). Table 2 presents the selected studies with their specifications. Eight out of the 11 studies showed a linkage between UA and improved prognosis in ALS patients, in which UA served as an antioxidant neuroprotective factor. However, in three studies, further research is required to prove the effect on the pathogenesis of the disease.

**Table 2:** Specifications of the included studies in this systematic review ALS: amyotrophic lateral sclerosis, UA: uric acid, ALSFRS-R: Amyotrophic Lateral Sclerosis Functional Rating Scale-Revised, DPR: disease progression rate, PEF: peak flow rate, A2AR: A2A receptor

Author	Journal	Publication year	Report/article type	Conclusion
Chen et al. [15]	Research Square	2021	Observational study	ALS patients have lower uric acid levels than the general population.
Chen et al. [16]	Research Square	2022	Observational prospective cohort study	To assess ALS patients' ALSFRS-R and DPR, uric acid may be a useful biomarker.
Haji et al. [17]	Clinical Neurology and Neurosurgery	2021	Meta-analysis	In ALS patients, serum UA levels may serve as a reliable predictive biomarker.
He et al. [18]	Frontiers in Public Health	2022	Observational cohort study	Low PEF, a predictor of respiratory impairment in ALS patients, was linked to low uric acid levels.
Kim [19]	Antioxidants	2021	Traditional review	Reduced urate levels could influence the onset and development of neurodegenerative illnesses such as ALS.
Mijailovic et al. [20]	Frontiers in Psychiatry	2022	Traditional review	At the beginning and/or progression of cognitive decline in various neurological illnesses, UA is thought to play a dual role as a pro- and antioxidant.
Mori et al. [21]	Biomedicine	2021	Traditional review	The activity of adenosine at A2ARs has been shown to play a function in pathogenesis and disease progression. Although there is evidence establishing that serum UA levels are associated with ALS progression as measured by the ALSFRS-R, more research is required to fully understand the effect. Uric acid is the end product of adenosine at the cellular level.
Tang et al. [22]	Frontiers in Neurology	2021	Observational cross-sectional study	In ALS patients, plasma uric acid may be used to determine the likelihood of cognitive impairment.
Wang et al. [23]	Oxidative Medicine and Cellular Longevity	2019	Systematic review and meta-analysis	In ALS patients, the levels of antioxidant uric acid were considerably decreased. This bolsters the scientific evidence that pro-oxidative imbalances contribute to the pathogenesis of ALS.
Xu et al. [24]	Frontiers in Neurology	2021	Longitudinal cohort study	Baseline serum uric acid levels are negatively correlated with mortality, particularly in males with ALS.
Han et al. [25]	Redox Report	2022	Retrospective observational study	After taking edaravone, the levels of serum uric acid could be utilized to monitor the course of ALS condition.

Study Type

We included six observational cohorts, one meta-analysis, one systematic review and meta-analysis, and three traditional reviews.

Included Disorders

All records and reviews were related to the effect of uric acid on the progression of ALS disease.

Discussion

We used this systematic review to help us learn more about the impact of serum UA on accelerating or delaying the course of ALS. This systematic review included 11 articles with a low risk of bias and good quality. Rules and ethical standards were carried out in this systematic review.

This systematic review has several benefits. The variety of study types included to cover all potential outcome points was one of this research's strengths. UA was the only putative biomarker that received attention because it exclusively affected ALS outcomes, and our review was unaffected by other indicators such as creatinine levels. To determine whether the UA effect is age-specific or not connected to a particular age group, the participants in the studies we selected ranged widely in age. This systematic review will focus on the progression of the disease and its correlation with UA levels as a reflection of the clinical course and severity of ALS.

Chen et al. published a prospective observational study in 2022 with 90 patients enrolled in the treatment arm and 80 in the control arm. Those patients were followed from January 2016 to August 2020 to examine the UA hypothesis for predicting neurological function and the ALS rate. Patients with a confirmed diagnosis of ALS, with lower motor neuron lesion evidence, aged 58±9.9 years old, were enrolled. Other comorbidities and impaired cognitive function were excluded. According to this study, there were significant variations in uric acid between the two groups (P=0.05); UA levels in the ALS group were lower than those in the control group. Chen et al. supported the theory that UA may be a reliable marker for assessing the Revised Amyotrophic Lateral Sclerosis Functional Rating Scale (ALSFRS-R) (measurement of ALS progression) [16]. Per our systematic review, we found fault with their study for not adding any new data on which to rely since their original study, which was conducted in 2021.

In 2022, the retrospective observational research by Han et al. examined the possibility that serum UA might be used to predict how ALS will develop after therapy with edaravone. Patient enrollment included 40 ALS patients who met the updated El Escorial criteria, had their baseline ALSFRS-R scores evaluated before starting their first cycle of edaravone medication, and their follow-up ALSFRS-R scores assessed 6-24 weeks later. The use of UA-lowering drugs, the existence of other comorbidities that might impair functional status, and other medical disorders that might affect UA levels were excluded. The study showed that patients with lower UA levels had considerably quicker ALSFRS-R/month than those with higher UA levels. The authors stated that following age correction, the presence of early symptoms, the use of riluzole, a high initial level of UA, and a slow rate of reduction in UA were linked to a slower course of the disease. In ALS patients on edaravone, the study found that high baseline values and a slow pace of UA drop may indicate a slower rate of disease progression [25]. According to our systematic evaluation, the study's weakness was not disclosing the precise number and location of the control group and neglecting to concentrate on the UA effect without edaravone. From our point of view, this study would add much information if it had focused only on UA.

He et al. published the outcomes of an observational cohort (retrospective and prospective) study in 2022. In this study, He et al. investigated the venous blood biomarkers related to respiratory function in ALS patients from southwest China. They used logistic regression to build prediction models based on those clinical biomarkers. Each participant met the updated El Escorial criteria for probable or certain ALS. Patients with other medical or neurological disorders and those with missing hematologic and biochemical baseline values were eliminated. Patients who had neoplastic disease manifestations were also disqualified. The study concluded that clinicians must assess the respiratory function status because most ALS patients in their advanced stages will experience respiratory impairment. The study revealed that lower UA levels were linked to lower peak flow rate (PEF) but were not independent predictors of PEF. Due to their concurrently declining levels of UA and creatinine, these patients ultimately have compromised respiratory function due to their low UA and creatinine levels [18]. Per our systematic review, the weaknesses of that retrospective cohort were the inability to focus on one biomarker, UA, which might be necessary for PEF risk. This study was the first to provide data regarding other diseases lowering or elevating UA levels in ALS patients. These modifications are recommended for future studies.

In 2021, Xu et al. concluded in a longitudinal observational cohort that, particularly for male ALS patients, there was an inverse correlation between baseline serum UA levels and risk of mortality. The enrollment criteria for that study included a mean age of onset was 55.7±11.2 years, and there were 207 male and 122 female patients. The study excluded renal conditions, cancerous tumors, and other illnesses that could impact survival rates and blood UA levels. The results showed that male ALS patients had UA concentrations that were significantly higher than those of female subjects, serum UA levels between patients with the bulbar onset and spinal onset were not significantly different, serum UA levels at baseline were positively correlated with body mass index, and, among male ALS patients, lower serum UA levels were significantly associated with shorter survival. Xu et al. concluded that, in the onset of ALS, UA seems to act as a preventative measure. Therefore, additional research is needed into the predictive significance of serum UA in ALS patients' illness severity, disease course, and survival. The association between UA levels and illness development was strongly inverse [24]. According to our comprehensive review, additional research is still required to examine UA levels and how they relate to the development of ALS over time. The authors of that study did not disclose any restrictions.

The importance of homocysteine, cystatin C, total cholesterol, triglycerides, creatinine kinase, and UA in developing ALS and neurological function was also examined in a 2021 observational study by Chen et al. In addition to 90 patients who served as the control group, 103 were enrolled. The inclusion criteria for these patients were an age of 58±9.9 years, El Escorial ALS clinical diagnostic criteria, and the absence of other comorbidities causing additional lower motor neuron disease or impaired cognitive function. The study showed significant variations in the amounts of UA between the two groups, with the ALS group having lower levels than the control group. The study found that ALS patients' UA levels were lower than those of healthy individuals, and the DPR of ALS and UA showed a negligible negative connection [15]. Per our systematic review, the study's flaw was that it did not concentrate on UA as a single biomarker.

Tang et al. reported the results of a 2021 cross-sectional investigation looking into using blood UA to predict cognitive decline in individuals with ALS. One hundred twenty-four subjects were enrolled in that study following the Edinburgh Cognitive and Behavioural ALS Screen (ECAS) and were categorized according to revised Strong's criteria. The patients were split into two sets; one set showed cognitive impairment (ALS-cie), and the other set was without cognitive impairment (ALS-ncie). UA levels were measured in both groups. Of the patients, 60% or more showed cognitive and/or behavioral impairment. The ALS-cie group had lower serum UA. Multivariate analysis showed that increased UA could predict a higher ECAS score. The study concluded that cognitive impairment was a common symptom in Chinese ALS patients. If education level and testing age are taken into consideration, plasma UA may be able to assess the likelihood of cognitive impairment in ALS patients. The study revealed that a lower plasma UA level was a reliable indicator of cognitive decline [22]. According to our systematic review, a direct connection between serum UA and the course of ALS was revealed in that study. Future research is needed to assess the effect of that biomarker on ALS patients worldwide, including people from other communities and the Chinese community.

In a traditional review by Kim in 2021, the author claimed, based on other investigators, that in vitro oxidative stress-induced toxicity is suppressed by urate treatment [19,26]. Additionally, it was discovered by other investigators that ALS patients had lower serum UA levels [9]. Therefore, decreasing urate levels could be linked to the development of neurodegenerative illnesses such as ALS. Kim claimed that in various neurodegenerative diseases characterized by the death of neuronal cells, a decrease in the nervous system's antioxidant tripeptide glutathione (GSH) appears to be a factor. The genesis and course of ALS are known to be influenced by compromised antioxidant defense mechanisms and the accumulation of oxidative damage brought on by increasing disruption in GSH homeostasis. Kim concluded that urate protects against injury to motor neurons by promoting the production of GSH [19]. The review's flaws included a hazy presentation of urate's involvement in raising glutathione levels and reducing oxidative stress in the central nervous system. Another drawback was that although the author admitted that GSH is a contributing component to ALS, the author asserted that additional research is still required to demonstrate that effect, completely denying the relevance of urea.

However, Mijailovic et al. (2022) conducted a traditional review of the effect of serum UA on the brain and cognitive functions in general. Mijailovic et al. showed that the review aimed to highlight the most recent research on the potential impact of UA on brain function and concentrate on oxidative stress and inflammatory processes as two possible molecular pathways for uric acid's harmful consequences. In the review, data was captured via PubMed and Web of Science with no restriction on the year of publication [20]. The review reported, based on other studies, that one of the most significant antioxidants in human plasma, UA, is a highly effective free radical scavenger [27]. They also highlighted that UA is protective in inflammatory central nervous system (CNS) processes. A claim was shown that exogenous administration of UA stopped blood-brain barrier (BBB) integrity breakdown and decreased its permeability to inflammatory cells. According to recent epidemiological research in the traditional review, UA levels were lower in ALS patients than in matched controls. High baseline UA levels were associated with slower disease progression and more prolonged survival. The traditional review concluded that UA appears to be a significant participant in the beginning and progression of cognitive decline in various illnesses, although many components that contribute to cognitive impairment have not yet been recognized. The pathophysiological function of UA and its clinical importance in affecting cognitive impairment are still under investigation. That may be partially explained by uric acid's dual nature and disparate characteristics and the wide range of unique diseases that can result in numerous constellations of dysfunctions in the cognitive domains [20]. In terms of analysis, the review highlighted the effect of UA on cognitive abilities and the clinical course of ALS and identified numerous pieces of research as evidence supporting that claim. The major problem with that review was the significant number of neurodegenerative diseases it covered and how little attention was paid to ALS, a fatal disease.

Mori et al., in 2021, revealed in a traditional review that there is a growing interest in the role of adenosine as a neuromodulator in ALS. Mori et al. aimed to investigate the potential of the A2A receptor (A2AR) as a target for ALS therapy and reviewed the potential pathways of chronic neurodegeneration via the adenosinergic system, the potential of UA as a biomarker, and the acute symptomatic pharmacology of A2A receptor antagonism (including phrenic motor facilitation). The review discussed the metabolism of adenosine in neural systems. Adenosine is broken down into inosine by the activity of adenosine ecto-deaminase (ecto-ADA), and clearance is carried out by non-concentrating nucleoside transporters. Adenosine kinase has been hypothesized to be predominant in neurons, whereas ADA has been hypothesized to be more prevalent in astrocytes. UA, the final human metabolite, is widely known to possess antioxidant characteristics. There is mounting data showing that blood UA levels correlate with ALS advancement as determined by the ALS Functional Rating Scale-Revised (ALSFRS-R), and serum UA has been proposed as a biomarker of ALS progression (particularly in the early phases). Mori et al. concluded that adenosine's action at A2ARs has been found to play a role in the pathophysiology and development of ALS. However, future studies are still needed to prove that claim [21]. The review's shortcoming was that it highlighted many adenosine properties without emphasizing UA and not stressing which studies have shown it to be the critical component in neuroprotective effects as it is the end product of adenosine.

In 2021, Haji et al. conducted a meta-analysis of hazard ratio-based studies that will be used to assess the value of UA as a predictive biomarker for ALS. They assessed the bias by visually evaluating the funnel plot and Egger's test and corrected it using a trim-and-fill method. According to the meta-analysis results, UA may be a predictive factor for ALS. The studies considered were uniform, and the reliability of these summary impacts was established by sensitivity analysis. A trim-and-fill procedure was used to correct for publication bias after it was discovered. According to the current meta-analysis, the authors concluded that serum UA levels in ALS patients might serve as predictive biomarkers [17]. According to our systematic review, the meta-analysis indicated statistically by numbers that UA might be a potential biomarker for ALS progression; nevertheless, the review was conducted only in a specific community, and the data analysis was carried out by authors rather than by another party to avoid the bias of the investigator. Our recommendation for future studies is to use a different party for statistical analysis.

Wang et al. (2019) conducted a systematic review and meta-analysis based on a claim that clinical studies' data have shown elevated levels of oxidative stress markers in the peripheral blood in ALS patients. However, the results were uncertain. They quantitatively investigated the blood levels of the designated oxidative stress indicators in ALS patients. They included papers examining blood oxidative stress marker levels in ALS patients and healthy controls after conducting a thorough search on PubMed and Web of Science. That was achieved by a random-effects meta-analysis that utilized 15 oxidative stress marker levels from 41 studies comprising 4,588 ALS patients and 6,344 control participants. The results showed that blood levels of glutathione and UA were considerably lower in ALS patients [23].

Wang et al. concluded that UA was markedly downregulated in ALS patients, and the results of other investigations as well as their collective clinical data on the malfunctioning of the antioxidant and oxidative systems in ALS patients supported the idea that oxidative stress plays a significant role in the development of the disease. The authors stated that their findings provided more insight into the blood oxidative stress marker profile of ALS patients and supported the clinical evidence that pro-oxidative abnormalities play a role in the etiology of the disease [23]. According to our systematic review, the study was one of the best at defining the function of UA as an antioxidant. To support the findings, data from numerous studies involving many people were collected and put through a meta-analysis. Their review needs to be more specific about the types of future studies they suggest should concentrate solely on the effects of UA and the mechanisms through which it exerts neuroprotective effects.

Limitations

The only limitation in this systematic review was the unavailability of articles focusing on the biomarker as the main factor.

## Conclusions

Our systematic review of the 11 selected articles examined the role of UA in ALS and its effect on the outcomes of that deadly disease. Our review of the selected articles found that a high level of UA was positively correlated with the clinical course and outcome of ALS. Additionally, UA is found at lower levels in healthy subjects than in ALS patients per those chosen articles. Its function as a potent antioxidant exerts a neuroprotective role in ALS patients per the articles with supportive evidence of that statement. Surprisingly, it is involved in many neurochemical interactions within the CNS. However, its definite impact is still under investigation and needs future research focusing only on ALS patients.

Future research may find it helpful to employ UA as a marker to track ALS's progression (outcome). Many illnesses, in addition to amyotrophic lateral sclerosis, may use biomarkers as prospective assessment and evaluation tools if research in this rapidly expanding field of study proves successful. The purpose of this systematic review was to address the impact of UA, which many experts believe has a significant influence on the course and outcome of ALS.
